# Experimental and Numerical Investigation on Oil Displacement Mechanism of Weak Gel in Waterflood Reservoirs

**DOI:** 10.3390/gels8050309

**Published:** 2022-05-17

**Authors:** Hongjie Cheng, Xianbao Zheng, Yongbin Wu, Jipeng Zhang, Xin Zhao, Chenglong Li

**Affiliations:** 1 Xinjiang Oilfield Company, Petrochina, Keramay 834000, China; chjie@petrochina.com.cn; 2 Research Institute of Petroleum Exploration & Development, Petrochina Daqing Oilfield, Daqing 163000, China; zhengxianbao@petrochina.com.cn (X.Z.); zhaoxin530@petrochina.com.cn (X.Z.); lcl716@126.com (C.L.); 3 Research Institute of Petroleum Exploration & Development, Petrochina, Beijing 100083, China; 4 School of Energy, China University of Geosciences (Beijing), Beijing 100083, China; 18152696912@163.com

**Keywords:** weak gel, oil recovery, numerical simulation, displacement efficiency

## Abstract

The production performance of waterflood reservoirs with years of production is severely challenged by high water cuts and extensive water channels. Among IOR/EOR methods, weak gel injection is particularly effective in improving the water displacement efficiency and oil recovery. The visualized microscopic oil displacement experiments were designed to comprehensively investigate the weak gel mechanisms in porous media and the numerical simulations coupling equations characterizing weak gel viscosity induced dynamics were implemented to understand its planar and vertical block and movement behaviors at the field scale. From experiments, the residual oil of initial water flooding mainly exists in the form of cluster, column, dead end, and membranous, and it mainly exists in the form of cluster and dead end in subsequent water flooding stage following weak gel injection. The porous flow mechanism of weak gel includes the preferential plugging of large channels, the integral and staged transport of weak gel, and the residual oil flow along pore walls in weak gel displacement. The profile-control mechanism of weak gel is as follows: weak gel selectively enters the large channels, weak gel blocks large channels and forces subsequent water flow to change direction, weak gel uses viscoelastic bulk motion to form negative pressure oil absorption, and the oil droplets converge to form an oil stream, respectively. The numerical simulation indicates that weak gel can effectively reduce the water-oil mobility ratio, preferentially block the high permeability layer and the large pore channels, divert the subsequent water to flood the low permeability layer, and improve the water injection swept efficiency. It is found numerically that a weak gel system is able to flow forward under high-pressure differences in the subsequent water flooding, which can further improve oil displacement efficiency. Unlike the conventional profile-control methods, weak gels make it possible to displace the bypassed oil in the deep inter-well regions with significant potential to enhance oil recovery.

## 1. Introduction

How to effectively shut in the high permeability channels and divert injected water to the bypassed low permeability regions, is a crucial question for the water flooding process in the late stage of water injection. After years of in-depth development in waterflood reservoirs, the residual oil is highly dispersed and mainly distributed in the deep area of the inter-well region [1], conventional modification of good pattern and injection and production parameters is less effective [2], and profile-control by traditional gels or polymers is no longer feasible as the high-concentration gels consolidate quickly and can only be effective in plugging the wellbore vicinity [3,4,5,6]. On the contrary, weak gels have a competitive edge over conventional gels in the following aspects: conventional gel has a relatively high concentration of polymer and crosslinker, high gel strength, short gel formation time, and high cost. Therefore, it can only be used for permeability adjustment in the near-well zone, but cannot effectively solve the problem of deep reservoir heterogeneity [6,7,8]. Weak gel, however, uses delayed crosslinking technology to inject a certain concentration of polymer and crosslinking agent into the deep reservoir, forming a polymer gel of a certain strength in the high permeability zone far from the injector vicinity, forcing the subsequent fluid to shift into the low permeability zone with high oil saturation, expanding the swept volume and improving the oil displacement efficiency [9,10].

Extensive laboratory investigation and field applications of weak gels have been performed in past years [7,8]. Different gel systems including ultra-high molecular weight HPAM/phenolic weak gel system, particle gels, gel agents with a high salt resistance, solvent–polymer weak gels, colloidal dispersion gels, et al., have been developed for oil reservoirs with various conditions [9,10,11,12,13,14], which effectively expand the applicability and potential of weak gels. Moreover, the hybrid process of weak gels with other recovery methods is also a new trend, such as the combination of weak gels and microorganisms, and the CO_2_-gel fracturing system in shale oil reservoirs [15,16].

Evaluation of gelation systems directly influences the type, operational parameters, and application performance of weak gels [17,18,19], in which numerical simulations were performed to study the influence factors of gelling performance [20,21], and laboratory experiments including NMR and coreflooding were used to investigate the displacement mechanisms [22,23,24].

However, the flow characteristics of the weak gel system in the porous media and the micro-mechanisms of oil displacement by reducing relative permeability of water phase in high permeability channels and expanding the water flooding swept area are not fully understood. The numerical simulation coupling experiment phenomenon and the equations characterizing migration dynamics of weak gel system in porous media, which describes the process of gel deformation and migration under the influences of pressure gradient, velocity, retention, time, and other factors, was yet to be performed previously. The visualized micro-scale experiments and the field scale numerical simulations were performed in this study to further investigate the oil displacement dynamics of weak gels and subsequent waterflooding, which is significant to guide the extensive application of weak gels.

## 2. Experiment

### 2.1. Experimental Equipment and Materials

The experimental oil is the simulated oil prepared using crude oil and kerosene in the laboratory, with a viscosity of 3.8 mpa·s at 45 °C. The experimental water uses the field-produced water with a salinity of 4800 mg/L. The weak gel used in the experiment is a polymer and chromium crosslinking agent (1600 mg/L × 2000 mg/L), in which the molecular weight of the polymer is 800 million. The experimental temperature is 45 °C to simulate the formation and reservoir conditions.

**Figure 1:**
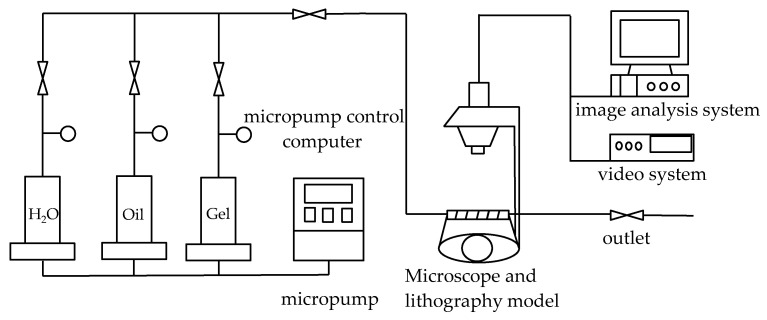
Visual connection diagram of micro-displacement experimental device.

Experimental instruments include the microlithographic glass model, magnetic stirrer, Brookfield viscosimeter, HW-2B thermostat with temperature control accuracy ±0.5 °C, electronic balance with sensing accuracy ±0.01 g, analytical balance with sensing accuracy ±0.0001 g, one micro-pump (range 0.5–10 mL/h), one micro-pump control computer, one high-magnification microscope, and one camera. The experimental equipment and process are shown in Figure 1.

### 2.2. Experimental Procedure

The experiments were carried out according to the following procedures:

1. Preparing the weak gel system required by the experiment: 0.4 g polymer was added into 200 mL produced formation water and stirred for 1 h. Then 0.9 g crosslinking agent and 0.2 g stabilizer were added to the solution and stirred for 1 h.
2. Filtering the crude oil to avoid blocking the model.
3. Saturating oil after the model is vacuumed.
4. Water flooding (constant speed 0.03 mL/h), observe the microscopic seepage process in the process of water flooding, and film the distribution and morphology of residual oil in the micro model until no oil is produced by water flooding.
5. Injecting 0.3 PV weak gel system into the lithography model while recording the microscopic oil displacement process by micro-camera.
6. Placing the lithographic model in a 45 °C incubator for 2 h, and wait for the weak gel system to gel.
7. The photolithography model was displaced by subsequent water until no oil was produced in the model. The microscopic oil flooding process was recorded by micro-camera.
8. Image analysis.
9. Cleaning the visualized model and preparing for the next experiment.

## 3. Analysis of Experimental Results

### 3.1. Phenomenon Description

#### 3.1.1. Oil Displacement Dynamics and Residual Oil Types in Water Flooding Stage

**Figure 2:**
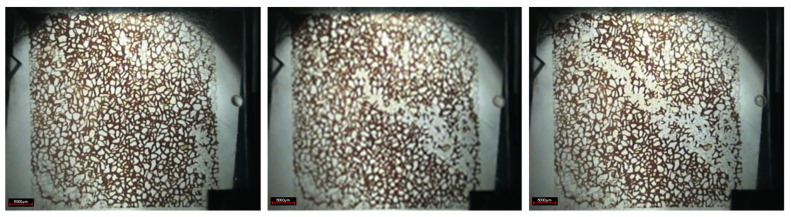
Water–oil displacement stage.

The oil displacement photos at different stages were recorded by micro-camera to describe the experimental phenomena. It is shown in Figure 2 that the injection end is in the lower right corner, and the production end is in the upper left corner. In the stage of water flooding, under the action of injection pressure at both ends of the photolithography model, the injected water communicates a curved water flow channel along the direction of the injection end and production end. The injected water front surges forward in the large channel area with low flow resistance, forming an obvious dominant channel, which is manifested as a fingering phenomenon. Due to the large flow resistance of injected water in the small pore, the small pore area with large flow resistance becomes the main enrichment area of residual oil.

**Figure 3:**
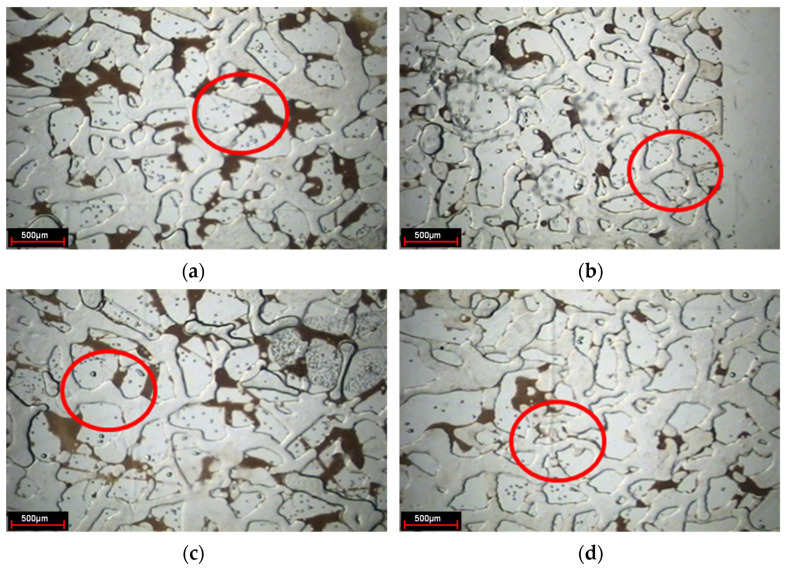
Distribution of residual oil after water flooding. (**a**) Cluster residual oil; (**b**) dead end residual oil; (**c**) columnar residual oil; (**d**) membranous residual oil.

After the water flooding stage, the residual oil in the pores and throats is evenly distributed along both sides of the dominant channel. The residual oil mainly exists in the form of cluster, column, dead end, and membrane, in which the cluster residual oil remains in small throat pore clusters surrounded by unobstructed large pores in water flooding, the columnar residual oil remains in isolated plug shape or columnar shape at the throat of connected pores, the dead end residual oil is isolated in the form of droplets in the dead angle of the pores where the injection water cannot sweep, and it is usually the residual oil with one end closed or one end very difficult to flow, and the membranous residual oil, as shown in Figure 3, is an oil film attached to the pores or inner wall of throats during water flooding.

#### 3.1.2. Oil Displacement Dynamics and Residual Oil Characteristics in Weak Gel Injection Stage

**Figure 4:**
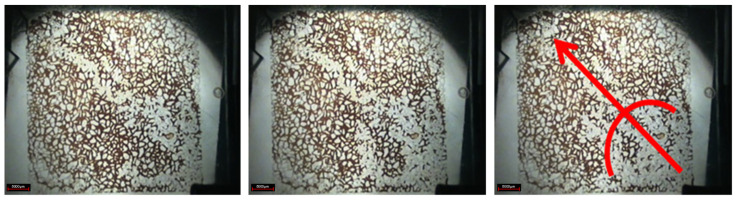
Weak gel injection stage (The arrow is the demonstration of aquauous weak gel displacement orientation, and the red arc is the swept area).

At the stage of weak gel injection, under the action of injection pressure, the weak gel front advances forwards in a circular shape, and the advance speed of the main streamline is faster. Weak gel preferentially migrates forward in the preferential flow channel generated in water flooding (Figure 4). In addition, in the areas on both sides of the water flow channel, the injected volume increased significantly, and the residual oil in the smaller channels that were not previously driven by injected water was also driven forward for a certain distance by the weak gel. The displacement front was relatively more uniform than water flooding, indicating that the weak gel played a role in adjusting the displacement profile.

Due to the influence of profile-control of weak gel, the residual oil near the injection end with high flow resistance was displaced. By the end of the weak gel injection, the residual oil mainly exists in the pores and throats close to the production end with high flow resistance and on both sides of the weak gel injection channel, presenting a relatively uniform distribution on the whole and relatively scattered distribution in local areas.

**Figure 5:**
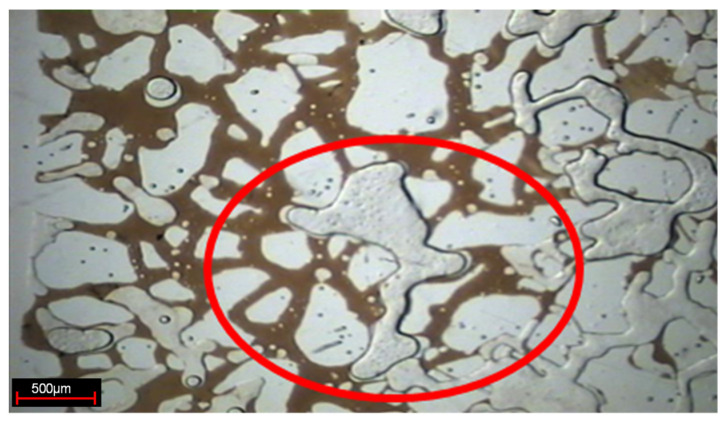
Weak gel group (The red circle is the weak gel group).

Changing the microscope lens and using the camera to take photos to observe the present form of a weak gel in the pores after injection, as indicated in Figure 5. In the subsequent water flooding stage, the weak gel system after gelation exists in the form of gel groups in the large pore channels, thus playing the role of selective plugging.

#### 3.1.3. Oil Displacement Dynamics and Residual Oil Characteristics in Subsequent Water Flooding Stage

**Figure 6:**
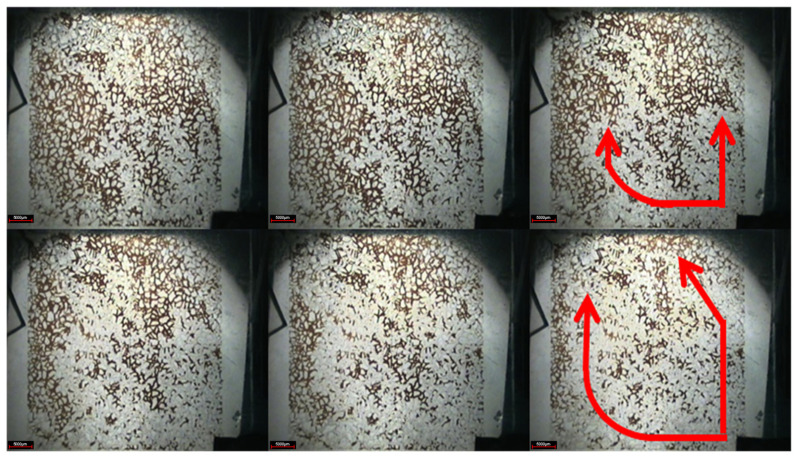
Subsequent water flooding stage (The arrow is the demonstration of subsequent water displacement orientation).

As shown in Figure 6, in the subsequent water flooding stage, due to the gelation of the weak gel system blocking the original water channel, the subsequent injection of water bypassed the original flow channel with the increase of water injection, and displaced the previously undeveloped oil in the small pores to the producing end, indicating that the weak gel plays a role in adjusting the displacement profile. Moreover, the injected water displaces the continuous weak gels along the pore extension direction, indicating that the weak gel also plays an oil displacement role. By comparing the pictures of the water flooding stage and subsequent water flooding stage, it can be seen that the sweep area of injected water has been significantly improved. A large number of residual oil in pores and throats is displaced by subsequent water, and the distribution of residual oil is very scattered. Microscopic observation shows that the residual oil mainly exists in the pore and throat in the form of clusters and dead ends after the subsequent water flooding stage.

#### 3.1.4. Description of Local Oil Displacement

**Figure 7:**
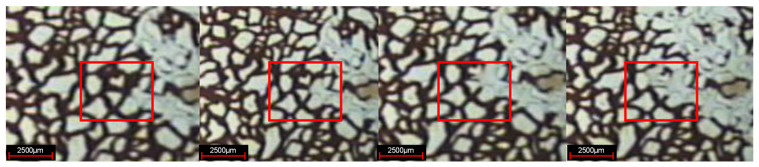
Oil displacement in water flooding stage.

**Figure 8:**
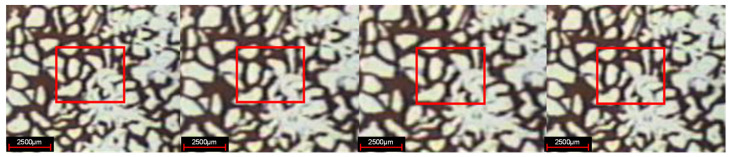
Oil displacement in weak gel injection stage.

**Figure 9:**
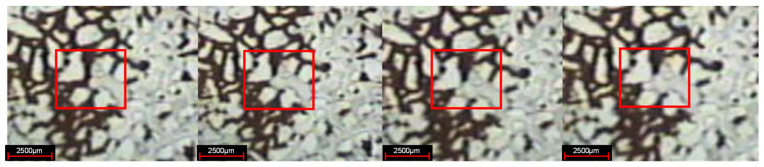
Oil displacement in subsequent water flooding stage.

In the experiment of weak gel microcosmic oil displacement, the phenomenon of oil displacement is different for parallel channels with different pore sizes in different oil displacement stages. In the stage of water flooding, for parallel channels with different sizes, the injected water preferentially replaces crude oil in larger channels, as shown in Figure 7. In the weak gel injection stage, for parallel channels with different sizes, the injected water preferentially replaces the crude oil in the smaller channels, as demonstrated in Figure 8. In the subsequent water flooding stage: for parallel channels with different sizes, the injected water preferentially replaces crude oil in larger channels, as indicated in Figure 9.

### 3.2. The Porous Flow Mechanism

The visualized microscopic oil displacement experiment of weak gel acting on the photoengraving model shows that weak gel first flows through the large pore channels during injection, so the subsequent injected water cannot pass through the large pore channels occupied by weak gel, then it is forced to flow to the pore channel not swept by the early water flooding. With the increase of injection water, the injected water pushes part of the weak gel forward, stops again when it meets small pores, and changes the direction of water flow to drive out the residual oil that has not been used in the stage of water flooding, improving the swept volume of injected water.

Through the analysis of the experimental results, the main mechanism of weak gel profile-control is obtained as follows: the preferential plugging of large channels, the integral and staged transport of weak gel, and the residual oil flow along pore walls in weak gel displacement.

1. Preferential plugging of large channels

In the phase of weak gel injection, with the increase of injected volume, at the injection end, the weak gel is displaced to the producing end in a circular shape. As the flow resistance of the gel in the high permeability area is less than that in the low permeability area, the main line direction of the weak gel is the direction of the high permeability area with low flow resistance and high porosity. After 2 h of the coagulation stage, the large pore channel is preferentially blocked.

**Figure 10:**
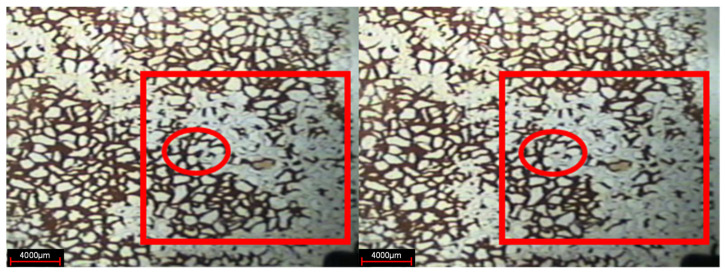
Preferential plugging of large channels.

As demonstrated in Figure 10, weak gel preferentially flows into large channels with low seepage resistance. The residual oil in the circle is driven by weak gel, indicating that the weak gel has preferentially entered the large pore channel along the water flow channel generated by water flooding oil.

2. The integral and staged transport of weak gel

As weak gel has a certain viscoelasticity, it mostly flows in the pore and throat structure in the form of gel groups in the seepage process, and the migration has strong integrity and coherence. Under the action of injection pressure, it is like a snake whose shape can change along the pore structure, advancing in the pore and throat. In addition, the migration of weak gel in the pore is phased. Before entering a certain pore, the weak gel accumulates liquid amount and pressure first. When the pressure reaches the threshold pressure to break through the pore, it immediately pushes into the pores.

**Figure 11:**
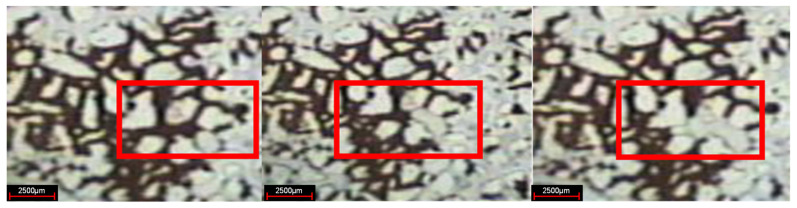
Weak gel transport.

As demonstrated in Figure 11, in the process of residual oil displacement, the weak gel pushes the residual oil forward as a whole, like a snake whose shape can change along the pore structure, and its migration has integrity and stages.

3. The residual oil flow along pore walls in weak gel displacement

Due to the viscoelasticity of weak gel and the integrity of its migration, the weak gel can migrate forward close to the pore wall when it migrates in the pores, so the residual oil in membrane form attached to the pore wall can also be displaced by weak gel, as shown in Figure 11 above.

### 3.3. The Profile-Control Mechanism

Through further analysis of the experimental results, the main mechanism of weak gel profile-control is obtained as follows: weak gel selectively enters the large channels, weak gel blocks large channels and forces subsequent water flow to change direction, weak gel uses viscoelastic bulk motion to form negative pressure oil absorption, and the oil droplets converge to form an oil stream.

1. The weak gel selectively enters the large pore channels

The main flow path of weak gel injected into the model is along the direction of low flow resistance and high porosity, which is often consistent with the path of water flooding in the early stage. As the weak gel injected into the model has a certain viscosity, it has a certain integrity and continuity in the flow process. However, due to the large size of the large pores, the shear rate of the weak gel is relatively low, so the weak gel can flow more smoothly while maintaining its integrity and continuity. Therefore, under a certain injection pressure, the weak gel will overcome the resistance along the path and migrate in the large channels with low resistance, as exhibited in Figure 10 above.

2. The gelation blocks the large pore channels and diverts the subsequent water flow direction

In the subsequent water flooding stage, the viscosity of weak gel after gelation is relatively high, which can form a blockage in pores and produce an end face effect, and the critical pressure entering small pores is much higher than that of oil and water. Therefore, under a certain pressure, the migration of weak gel is difficult, and the probability of entering small pores is very small. According to the microscopic experimental results, in the subsequent water flooding process, due to the blocking phenomenon of weak gel in the channels under a certain pressure, the liquid flow of injected water is forced to change direction and flow to the small-channel area with relatively small resistance, so that the residual oil that is undeveloped in the stage of water flooding is displaced and the swept volume is increased.

**Figure 12:**
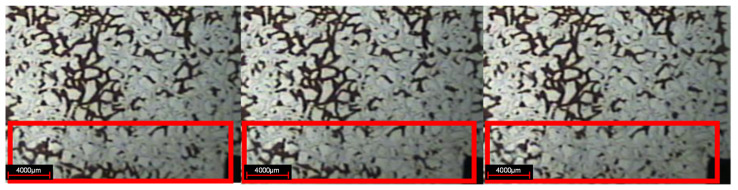
Subsequent water flow redirection.

As shown in Figure 12, in the subsequent water flooding stage, due to the weak gel after gelation blocked the pores, the subsequent injection of water is forced to flow into the small pores to displace the residual oil, which plays a role in adjusting the water imbibition profile.

3. The viscoelastic gelation moves integrally to absorb oil by negative pressure

For the weak gel, the chemical structure of the cross-linked polymer molecular coils is relatively stable, and the stability after gelation is better, which mainly reflects good viscoelasticity, integrity, and coherence during migration, and obvious water–glue interface with subsequent water. When the injection pressure reaches a certain level, the weak gel group flows through the hole like a snake through the grass, and flows rapidly in the direction of relatively little resistance. Due to the instantaneous speed of the integral migration of the weak gel group, and its good viscoelasticity, integrity, and coherence during migration, the subsequent fluid cannot be filled in time, resulting in instantaneous ‘negative pressure’ (the pressure of the main channel is lower than that of the surrounding channels). At this point, the oil or water in the surrounding porous channel overcomes the internal threshold pressure and extrudes out of the porous channel and is sucked into the oil stream.

**Figure 13:**
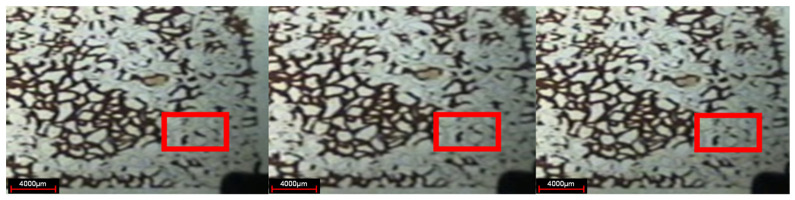
Negative pressure oil absorption.

Figure 13 indicates that in the weak gel injection stage, negative pressure is formed due to the viscoelastic bulk movement of the weak gel, which sucked out the residual oil in the subsequent water flooding stage.

4. Oil droplets converge to form oil stream

**Figure 14:**
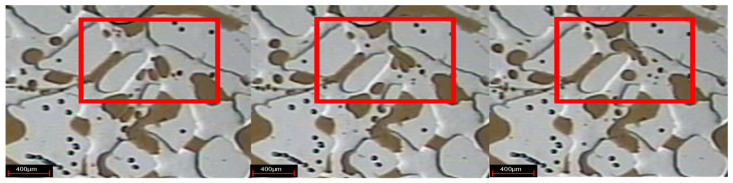
Accumulation of oil droplets into oil stream.

In the microscopic displacement experiment, it is also found that in the process of weak gel displacement, a large amount of oil in small pores is displaced, and gradually accumulates into oil droplets in large pores, which move forward and form continuous oil droplets. The thickness of oil droplets thickens with the displacement, and finally, they migrate to the outlet in the form of continuous oil flow. As shown in Figure 14, three small oil droplets converge to form a large oil droplet when passing through a narrow throat and migrate forwards in the form of an oil stream.

## 4. Numerical Simulation

### 4.1. Simulation Model Parameters

The numerical simulation is carried out using the CMG-STARS reservoir numerical simulator. The model is established using a Cartesian grid system, which is divided into 19 × 19 grids in X and Y directions with a grid size of 20 m, and 10 grids in the Z direction with each grid thickness of 10 m. The five-point well pattern was used which includes one injector in the center and four corner producers. The basic parameters and their values of the sector model are shown in Table 1.

**Figure 15:**
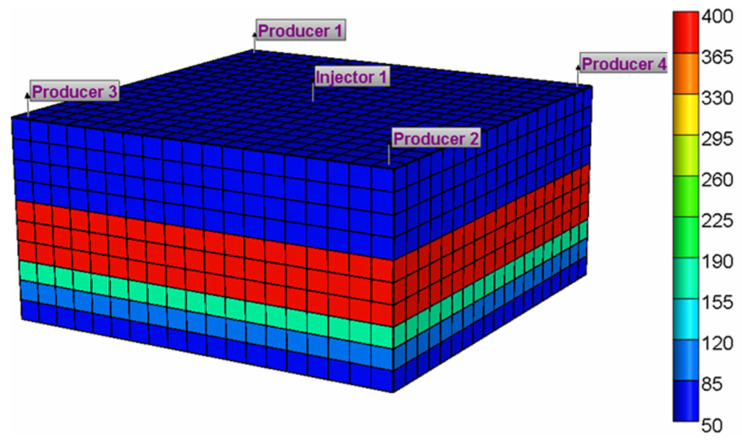
A 3D numerical model of reservoir.

In this model, the gel injection rate is 0.097 PV/a, the maximum injection pressure is 20 MPa, the minimum bottomhole flow pressure is 3 MPa, and the cumulative gel injection volume is 0.29 PV. The basic data required for reservoir fluid modeling are shown in Table 2. The established 3D numerical model is demonstrated in Figure 15.

### 4.2. Equations Influencing Weak Gel Displacement Dynamics

The efficiency of water flooding is largely related to the mobility ratio of displacing and displaced fluids. Definition of mobility ratio:

(1) M=Displacing phase mobilityDisplaced phase mobility=kwμwkoμo=kwko×μoμw

where, $k_{w}$—Relative permeability of water; $\mu_{w}$—Viscosity of water; $k_{o}$—Relative permeability of oil; $\mu_{o}$—Viscosity of oil.

Due to the high mobility ratio, the injected water moves faster than the displaced oil, resulting in fingering. The injected water bypassed the displaced oil and migrated toward the producing well. Most of the oil is unswept by the water because of fingering, which is the water flow channel toward the producing well. Once the water channel is formed, water will bypass residual oil in the reservoir and flows directly from the injection well to the producing well.

After the injection of weak gel, the liquid viscosity can be calculated according to the nonlinear mixing Equation (1):

(2) $$Ln\left(\mu_{\alpha }\right)=\sum_{i=1}^{n_{c}\in s}f\left(f_{\alpha i}\right)\times Ln\left(\mu_{\alpha i}\right)+N\times \sum_{i=1}^{n_{c}\notin s}f_{\alpha i}\times Ln\left(\mu_{\alpha i}\right)$$

where, $\mu_{\alpha }$—Mixed viscosity of aqueous phase (α= w) or oil phase (α= o); $\mu_{\alpha i}$—Viscosity of aqueous phase (α= w) or oil phase (α= o) component “i”; $f_{\alpha i}$—Weight factor of non-critical component “i” in nonlinear mixing calculation of aqueous phase (α= w) or oil phase (α= o); $f\left(f_{\alpha i}\right)$—Weight factor of key component “i” in nonlinear mixed calculation of aqueous phase (α= w) or oil phase (α= o); $n_{c}\in s$—Key group fraction in liquid phase; $n_{c}\notin s$—Scores of other groups except the key group; N—Normalized factor.

**Figure 16:**
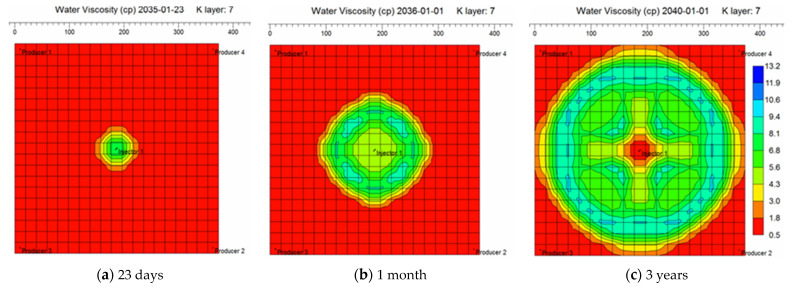
Aqueous phase viscosity distribution at different stages of weak gel injection.

By incorporating the equations above characterizing injected liquid viscosity and mobility ratio induced migration dynamics of weak gel system in porous media into the simulation model, it is able to simulate gel deformation and migration dynamics under the influences of pressure gradient, velocity, retention, time and other factors. The aqueous phase viscosity distribution field maps in different periods of weak gel injection are given through numerical simulation, as exhibited in Figure 16. The simulation results indicate that weak gel can increase the viscosity of the aqueous phase, thus reducing the mobility of water, reducing the fingering phenomenon, improving the plane heterogeneity, and enhancing the recovery factor.

### 4.3. Dynamics of Weak Gel Injection

**Figure 17:**
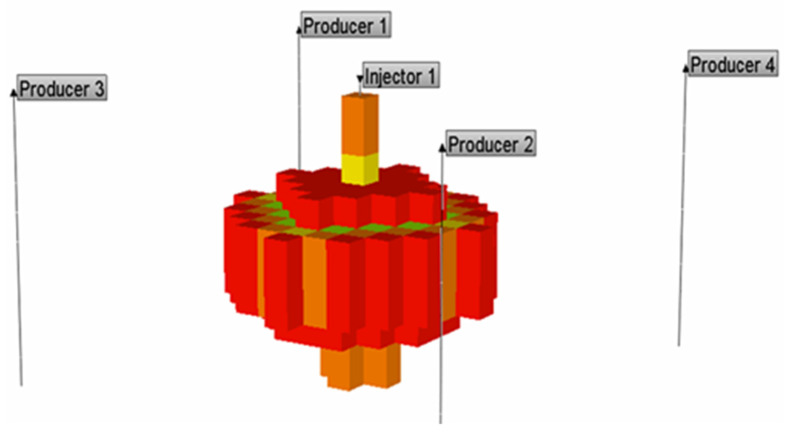

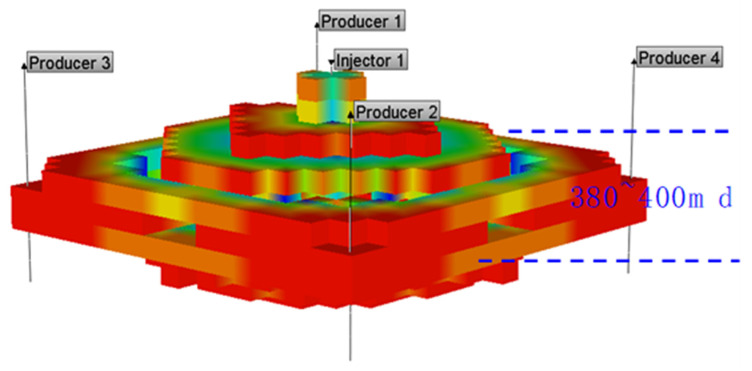
A 3D distribution of weak gel mole fraction.

As shown in Figure 17, numerical simulation results demonstrated that the higher the permeability, the lower the flow resistance, so the weak gel will preferentially enter the high permeability layer and increase its flow resistance. Consequently, the subsequent injected water will enter the low permeability layer or the low permeability area and expand the swept volume.

**Figure 18:**
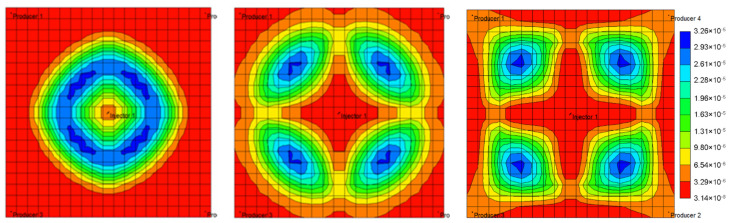
Planar distribution of weak gel mole fraction.

According to the numerical simulation results, the plane distribution of weak gel mole fraction in Figure 18 shows that the main route of weak gel flow is along the direction of the high permeability flow channel, which is often the channel of water breakthrough in the early waterflood stage. After weak gel profile-control flooding, water pushes the weak gel to flow forward. Therefore, weak gels continuously expand the sweep region and displace the residual oil in this region to the producing well, so weak gels play two roles adjusting the heterogeneity and displacing oil.

### 4.4. Diversion of Subsequent Water Flooding

**Figure 19:**
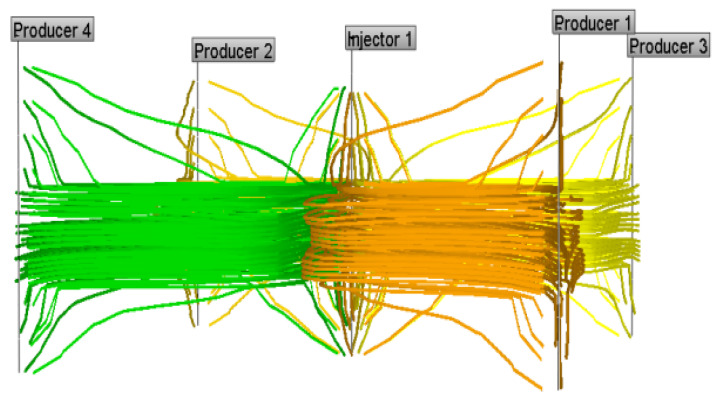
A 3D streamline distribution of initial water flooding.

**Figure 20:**
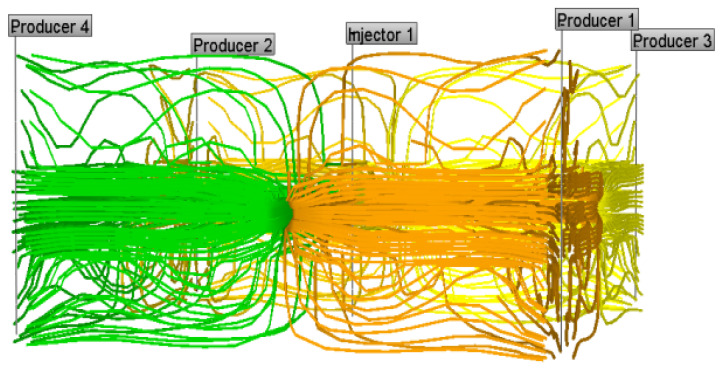
A 3D streamline distribution of subsequent water flooding.

Based on the established injection-production system of a five-point well pattern, the comparison of three-dimensional streamline distribution of initial water flooding and subsequent water flooding following weak gel was simulated numerically. It can be seen from Figure 19 that in the process of water flooding, injected water mainly flows along the highly permeable layer, and weak gel first enters the large pore originally occupied by water after injection. Under the effect of subsequent injection water, the weak gel continues to move forward along the large pore channels with low resistance. Meanwhile, the presence of weak gel increases the flow resistance of the large pore channels, forcing the injection water to change direction. Figure 20 indicates that the subsequent injected water can enter the low permeability zone unswept in the initial water injection, thus improving the sweep efficiency and ultimate recovery of the waterflood.

**Figure 21:**
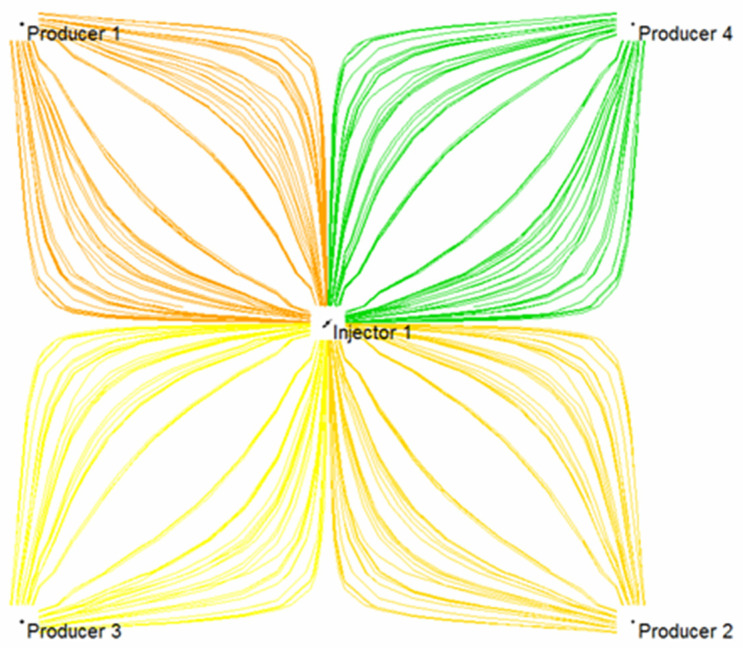
Planar streamline distribution of initial water flooding.

**Figure 22:**
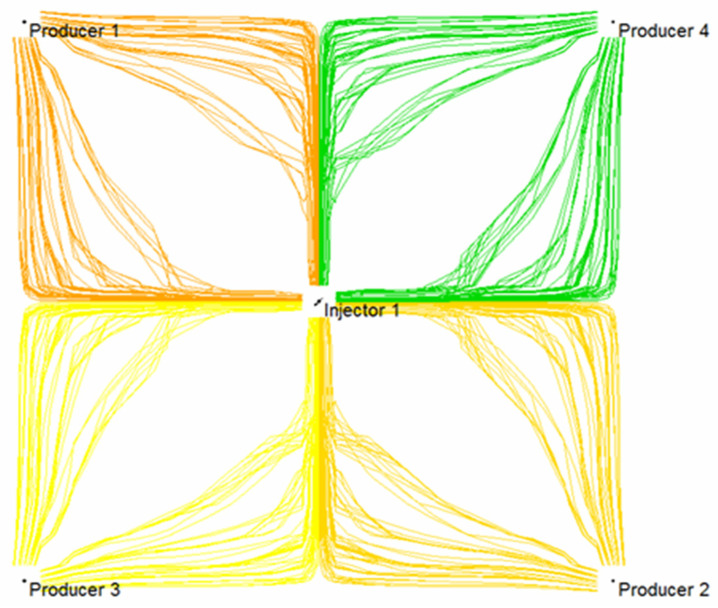
Planar streamline distribution of subsequent water flooding.

The comparison of the planar streamline distribution in Figure 21 and Figure 22 shows that weak gel will change the flow direction of other fluids when it flows in porous media. The reservoir itself has macroscopic and microscopic heterogeneity, and microscopic heterogeneity is mainly reflected in the difference in pore size, pore distribution, and pore surface properties in the rock pore structure. Therefore, in the process of water flooding, the injected water always tends to enter the large pore channels with low resistance, and the residual oil always remains in the small pores or in the form of oil droplets in the center of the large pores. According to channel flow theory, when the oil phase loses its continuity, it becomes residual oil and cannot be recovered. After the weak gel is injected into the porous media, the flow direction of the subsequent injected water can be diverted, as shown in Figure 22, forcing the injected water to enter the unswept area, thus improving the sweep efficiency of the subsequent water flooding.

## 5. Conclusions

1. By analyzing the distribution patterns of residual oil in pores and throat through visualized microscopic oil displacement experiments at different stages, the residual oil of initial water flooding mainly exists in the form of cluster, column, dead end, and membranous, and it mainly exists in the form of cluster and dead end in subsequent water flooding stage following weak gel injection.
2. The porous flow mechanism of weak gel includes the preferential plugging of large channels, the integral and staged transport of weak gel, and the residual oil flow along pore walls in weak gel displacement.
3. The profile-control mechanism of weak gel is as follows: weak gel selectively enters the large channels, weak gel blocks large channels and forces subsequent water flow to change direction, weak gel uses viscoelastic bulk motion to form negative pressure oil absorption, and the oil droplets converge to form an oil stream, respectively.
4. Numerical simulation coupling equations characterizing weak gel viscosity induced dynamics indicate that weak gel can effectively reduce the water-oil mobility ratio, preferentially block the high permeability layer and the large pore channels, divert the subsequent water to flood the low permeability layer, and improve the water injection swept efficiency. A weak gel system is able to flow forward under high-pressure difference, which can further improve oil displacement efficiency besides flow diversion.

## Figures and Tables

**Table 1:** Basic data of the geological model.

Reservoir Parameters	Parameter Value
Reservoir top depth (m)	1400
Permeability (mD)	50, 55, 60, 80, 380, 400, 390, 180, 100, 50
Porosity (%)	0.23
Original formation pressure (MPa)	16

**Table 2:** Basic data of the fluid model.

Reservoir Parameters	Parameter Value
Underground oil viscosity (mPa·s)	8
Surface crude oil density (kg/m^3^)	860
Water viscosity (mPa·s)	0.5
Oil volume coefficient (m^3^/m^3^)	1.09

## Data Availability

The data of this article is available on request through e-mail.
